# Annual Acoustic Presence of Fin Whale (*Balaenoptera physalus*) Offshore Eastern Sicily, Central Mediterranean Sea

**DOI:** 10.1371/journal.pone.0141838

**Published:** 2015-11-18

**Authors:** Virginia Sciacca, Francesco Caruso, Laura Beranzoli, Francesco Chierici, Emilio De Domenico, Davide Embriaco, Paolo Favali, Gabriele Giovanetti, Giuseppina Larosa, Giuditta Marinaro, Elena Papale, Gianni Pavan, Carmelo Pellegrino, Sara Pulvirenti, Francesco Simeone, Salvatore Viola, Giorgio Riccobene

**Affiliations:** 1 Dipartimento di Scienze Biologiche e Ambientali, University of Messina, Messina, Italy; 2 Istituto Nazionale di Fisica Nucleare (INFN) - Laboratori Nazionali del Sud, Catania, Italy; 3 Istituto Nazionale di Geofisica e Vulcanologia (INGV), Roma, Italy; 4 EMSO Interim Office c/o Istituto Nazionale di Geofisica e Vulcanologia, Roma, Italy; 5 Istituto Nazionale di Astrofisica - Istituto di Radioastronomia (INAF-IRA), Bologna, Italy; 6 Bioacoustics Lab, IAMC Capo Granitola, National Research Council, Torretta Granitola (TP), Italy; 7 Centro Interdisciplinare di Bioacustica e Ricerche Ambientali (CIBRA), Dipartimento di Scienze della Terra e dell’Ambiente, University of Pavia, Pavia, Italy; 8 Istituto Nazionale di Fisica Nucleare (INFN) - Bologna, Bologna, Italy; 9 Dipartimento di Fisica e Astronomia Università di Bologna, University of Bologna, Bologna, Italy; 10 Istituto Nazionale di Fisica Nucleare (INFN) - Roma1, Roma, Italy; Virginia Commonwealth University, UNITED STATES

## Abstract

In recent years, an increasing number of surveys have definitively confirmed the seasonal presence of fin whales (*Balaenoptera physalus*) in highly productive regions of the Mediterranean Sea. Despite this, very little is yet known about the routes that the species seasonally follows within the Mediterranean basin and, particularly, in the Ionian area. The present study assesses for the first time fin whale acoustic presence offshore Eastern Sicily (Ionian Sea), throughout the processing of about 10 months of continuous acoustic monitoring. The recording of fin whale vocalizations was made possible by the cabled deep-sea multidisciplinary observatory, “NEMO-SN1”, deployed 25 km off the Catania harbor at a depth of about 2,100 meters. NEMO-SN1 is an operational node of the European Multidisciplinary Seafloor and water-column Observatory (EMSO) Research Infrastructure. The observatory was equipped with a low-frequency hydrophone (bandwidth: 0.05 Hz–1 kHz, sampling rate: 2 kHz) which continuously acquired data from July 2012 to May 2013. About 7,200 hours of acoustic data were analyzed by means of spectrogram display. Calls with the typical structure and patterns associated to the Mediterranean fin whale population were identified and monitored in the area for the first time. Furthermore, a background noise analysis within the fin whale communication frequency band (17.9–22.5 Hz) was conducted to investigate possible detection-masking effects. The study confirms the hypothesis that fin whales are present in the Ionian Sea throughout all seasons, with peaks in call detection rate during spring and summer months. The analysis also demonstrates that calls were more frequently detected in low background noise conditions. Further analysis will be performed to understand whether observed levels of noise limit the acoustic detection of the fin whales vocalizations, or whether the animals vocalize less in the presence of high background noise.

## Introduction

The fin whale (*Balaenoptera physalus*) is considered to be the only mysticete species regularly present in the Mediterranean Sea [1, 2]. Here, fin whales constitute a genetically distinct population, which is widespread across the entire ocean basin [1–4]. In recent years, an increasing number of visual, acoustic and aerial surveys have confirmed the regular occurrence of fin whales in highly productive regions of the Mediterranean Sea, such as the protected area of the Pelagos Sanctuary for Mediterranean Marine Mammals [1, 5–8], in the north-western part of the basin. Recently, passive acoustic observations, reported by Castellote et al. [2, 9], revealed a seasonal movement of fin whales between summer and winter feeding grounds, from the Corso-Ligurian Basin to southern Spain and to the north African coasts. A partial seasonal exchange of individuals moving from the North Atlantic Ocean towards the Alboran Sea, across the Strait of Gibraltar, was also discovered using bottom-mounted audio recorders [2] and stable isotope analysis [10]. Despite this, very little is known about the routes that the population seasonally follows within the basin as a whole, nor in the Ionian Sea in particular. So far, the occurrence of fin whales off the eastern coast of Sicily has been poorly described, since only sporadic opportunistic observations were made during summer months in the region [1, 11]. Aïssi et al. [12] report the presence of solitary animals and small aggregations (1 to 3 individuals), both traveling and feeding, in the Strait of Messina. Sightings have occurred in different seasons and a regular autumnal movement of the animals between the eastern and western Mediterranean regions, across the Strait of Messina, has also been hypothesized [12]. Furthermore, several visual surveys [13–15] demonstrated that the area around Lampedusa Island (off the southwest coasts of Sicily) is an important feeding ground for the species. Here, fin whales have been frequently observed during the late winter and early spring months, engaged in surface feeding activities [1, 13–15]. Little information exists regarding the presence of fin whales around the Island of Malta during summertime [16]. Nevertheless, exhaustive reports about fin whale presence and acoustic activity offshore Eastern Sicily are not available prior to this study.

The main aim of this work is to investigate the presence of fin whales in the region off the eastern coast of Sicily by means of continuous passive acoustic monitoring. Acoustic detection may be influenced by a wide variety of factors, including source level variations, ambient noise, sound transmission path and distance between the emitter and the receiver [17]. The source level of fin whale vocalizations varies depending on their biological function [18]; hence, no universally accepted estimate exists for the species. It has been estimated in 186 dB re 1 *μ*Pa at 1 m [19], 159–184 dB re 1 *μ*Pa at 1 m [18], 189 ± 4 dB re 1 *μ*Pa at 1 m [20], 189 ± 5.8 dB re 1 *μ*Pa at 1 m [21]. High levels of anthropogenic noise are also expected in the area [22], since maritime traffic is highly intense offshore Eastern Sicily. This study presents a preliminary investigation into the possible correlation between background noise levels and acoustic detection of fin whale.

### Study area

**Fig 1:**
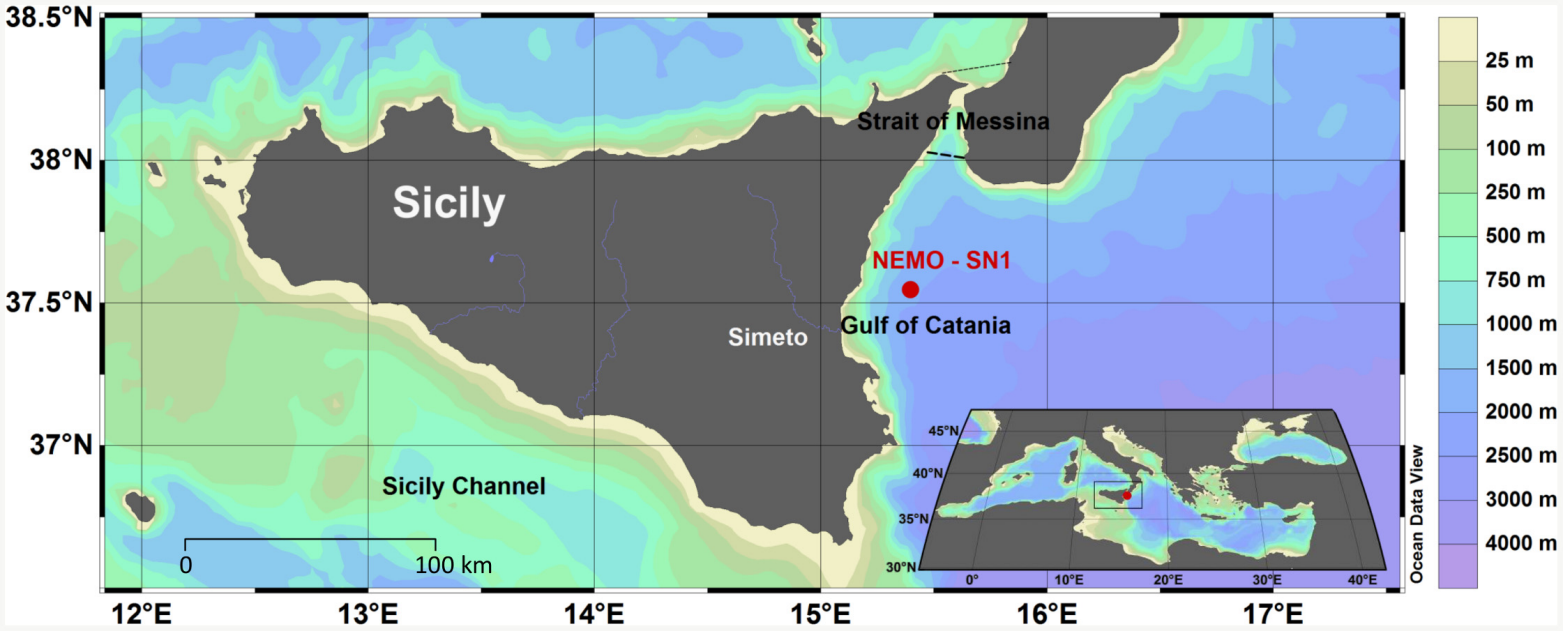
Study area. Bathymetric map of the region showing the geographic location [36] of the NEMO-SN1 deep-sea multidisciplinary cabled observatory, operative node of the EMSO Research Infrastructure.

The NEMO-SN1 deep-sea multidisciplinary observatory currently operates in the Gulf of Catania (37.54765 N, 15.3975 E), at a depth of 2,100 m [23, 24]. The northern side of the Gulf is characterized by steep slopes, where a depth of 200 m is reached in less than 2 km from the coast (Fig 1), whereas the Gulf’s most prominent features are the high geomorphologic and oceanographic heterogeneities. The presence of several river mouths (including the Simeto River, which has the biggest discharge in Sicily) determines a cyclic increase in nutrients, resulting in the growth of primary productivity, with peaks in spring and autumn. A secondary increase in water productivity is given by the currents involved in water circulation along the Strait of Messina, located over the northern side of the Gulf of Catania (Fig 1). The Strait connects the Ionian and the Tyrrhenian Seas and it includes a stable upwelling system, driven by strong tidal currents flowing between the two basins [25–27]. Furthermore, the presence of the euphausiid species *Meganictiphanes norvegica* has been observed year-round both in the Strait of Messina and along the Ionian Sicilian coasts [12, 14, 28, 29]. This species plays an important role in the diet of Mediterranean fin whales [14, 15, 30, 31], along with another euphausiid species, *Nictiphanes couchi* [15]. Deep waters and periodic increases in water productivity render the Gulf of Catania a suitable habitat for many cetacean species, including dolphins (*Delphinidae*), the sperm whale (*Physeter macrocephalus*) and the fin whale itself [32–35], which has been previously observed in this region, whilst involved in feeding activities during the spring months [14].

### Acoustic monitoring of fin whale vocalizations

Acoustic methods represent a well-established tool in cetacean conservation in the Mediterranean Sea [2, 33, 37, 38]. Cabled deep-sea multidisciplinary observatories, such as NEMO-SN1, extend cetacean monitoring capabilities [39, 40] by providing continuous real-time acoustic data for long-term observations [23, 32, 34, 35, 41, 42]. The fin whale acoustic repertoire is mainly composed of vocalizations emitted for intraspecies communication, at frequencies close to the lower limit of the human hearing range; these are referred to as “calls”. The best known fin whale vocalizations are called *20 Hz pulses*, calls centered around 20 Hz, used in long-range communication [19, 21, 43–45]. These sounds can be produced in short, isolated series or in long, stereotyped sequences called “songs” or “bouts” [19]. Songs are usually structured into complex patterned series and seem to be produced by male individuals, with a display function during courtship [46]. Songs may last for hours, alternated by resting periods of variable length [19, 45–48]. Calls with a frequency higher than 30 Hz have been less frequently reported and have been associated with short distance communication and geographic differences in populations repertoires [40, 45, 47, 49, 50]. The Mediterranean fin whale acoustic repertoire has been studied in the past in the western part of the basin and the most reported vocalizations may be grouped into two main call types [1, 2, 51, 52]:

- type “A”, also known as “classic pulse”, a downsweep signal from 23 to 17 Hz, which lasts about 1 second;
- type “B”, often called “back-beat”, with a constant frequency from 18 to 20 Hz, lasting about 0.8–1 second.

## Methods

### Acoustic data acquisition and analysis

**Fig 2:**
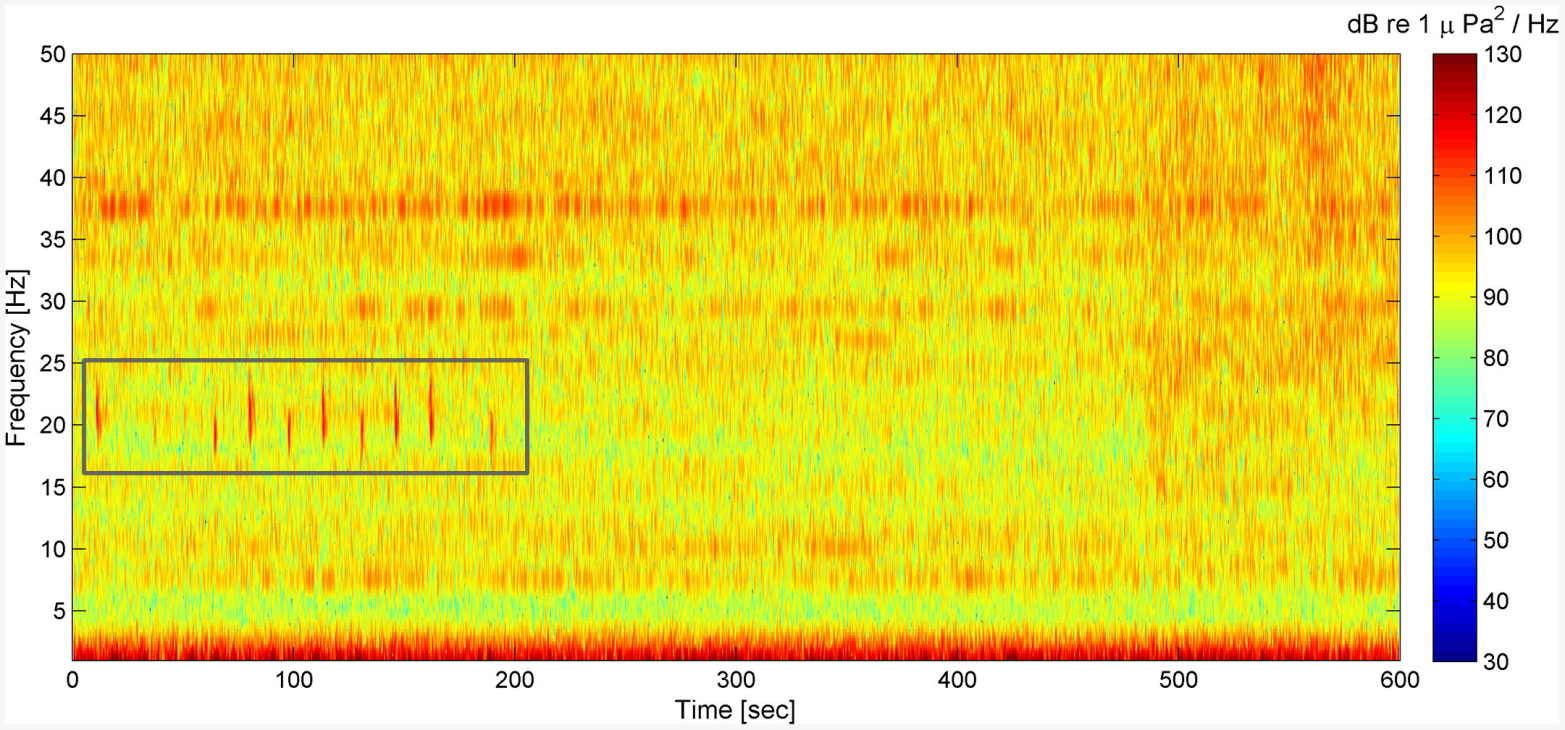
Typical analyzed spectrogram. A typical spectrogram (8192 points FFT, 4096 points Hanning window, 97% overlap) of a 10-min recording, computed and analyzed by expert operators. A sequence of 20 Hz calls, here included for clarity in a grey box, has been visually identified in this file.

Fin whale acoustic presence was revealed by analyzing the low-frequency acoustic data acquired through the NEMO-SN1 deep-sea cabled multidisciplinary observatory [23, 24]. This observatory is jointly operated by INGV (Istituto Nazionale di Geofisica e Vulcanologia) and INFN (Istituto Nazionale di Fisica Nucleare) within the activities of EMSO [42, 53] and KM3NeT (KM^3^ Neutrino Telescope) [54, 55] Research Infrastructures; the data acquisition system was designed and operated under the SMO (Submarine Multidisciplinary Observatory) project [56–58]. NEMO-SN1 is located 25 km, about 25 km off the Catania harbor, at the depth of 2,100 m [23] and it is equipped with several geophysical, oceanographic and acoustic sensors, including two low-frequency hydrophones [23]. Data used for this study were continuously recorded by the low-frequency hydrophone, model SMID DT405D(V)1, between July 2^nd^, 2012 and May 10^th^, 2013. The active component of the hydrophone is a piezoelectric omni-directional ceramic, which has a sensitivity of 197 ± 1 dB re 1V/*μ*Pa. The analogue signal was read on 2 channels, with an amplification factor of 30 and 30+30 dB respectively. The analogue signals were sampled at 2 kHz rate by two 12-bit A/D converters. This configuration allowed a nominal digital sensitivity @ 1 kHz of about 0.55 Pa (115 dB re 1 *μ*Pa), for the low gain channel, and of 0.017 Pa (85 dB re 1 *μ*Pa), for the high gain channel, while the dynamic range was 66 dB for each channel. Accordingly, the full range amplitudes @ 1 kHz were 1120 Pa (181 dB re 1 *μ*Pa) and 35 Pa (151 dB re 1 *μ*Pa), for the low and high gain channels, respectively. The digital hydrophone has a flat frequency response in the range from 50 mHz to about 1 kHz [59]. The time synchronization (date and time in ISO 8601: UTC notation, 24-h format) for the detected signal was provided by a GPS signal, distributed from the shore station to the observatory, used to label the digital acoustic data. The data, digitized offshore, were continuously (24/7) sent to the shore station through a 28 km electro-optical cable. On shore, the acoustic data stream was acquired and stored in 10-min files of a proprietary format. MATLAB code was developed to automatically convert each raw data file in standard WAV format with accurate time stamp included in the filename. This code was used to calculate the signal Power Spectral Density (PSD) and to generate the spectrogram of each 10-min recording (8192 points FFT, 4096 points Hanning Window, 97% overlap), assigning a different color to each amplitude value (dB re 1 *μ*Pa^2^/Hz). The image of the spectrogram in the 1–50 Hz frequency band was then automatically saved by the same code in a PNG image (Fig 2) for subsequent visual analysis, by expert human operators. Looking at the images of the spectrograms, the operator classified the signals as *20 Hz calls* when they simultaneously satisfied the following conditions:

1. duration from 0.5 to 1.5 seconds [19, 51];
2. frequency range between 15 and 30 Hz [19, 43, 51];
3. PSD exceeding 105 dB re 1 *μ*Pa^2^/Hz;
4. at least two signals within an interval from 6 to 46 seconds (typical duration of the Inter Call Interval for fin whale 20 Hz calls [19, 51]).

The PSD of the detected signals was evaluated by the operator, looking at the color scale of the spectrograms (Fig 2). The number of 20 Hz calls was assessed for each 10-min file and the corresponding hour in 24, 1-h time periods based on UTC time notation, was considered as detection hour. Following Stafford et al. [60] and Wiggins et al. [61], the number of detections/h was measured in four different light periods to evaluate the eventual occurrence of diel patterns in the detection rate. Begin and end times for sunrise, sunset and nautical twilight were obtained for the analyzed months, at the study location, from the United States Naval Observatory Astronomical Applications Department Web site [62]. The known time of each 10-min audio file was used to include the detections into these four light periods defined by the U.S. Naval Observatory: dawn, day, dusk and night [62]. Since data were not normally distributed (failed Lilliefors test for *p-value* < 0.01), the Kruskal-Wallis test was conducted for a non-parametric analysis of variance (ANOVA). This analysis was performed to ascertain whether the number of detections/h was constant over the four diel periods.

### Background noise analysis

The median values and percentile distribution of noise (PSD) were measured, as useful indicators of the typical background noise trends in the area [63, 64]. In particular, the frequency band between 17.9 and 22.5 Hz was selected for noise analysis, since it corresponds approximately to the one-third octave band centered at the frequency of 20 Hz, where all fin whale signals are emitted [19]. The integral noise amplitude value (dB re 1*μ*Pa) in this frequency interval was measured by software written in MATLAB every 0.5 seconds (sampling frequency 2 kHz, 2048 FFT points). The 5^th^, the 50^th^ and the 98^th^ percentiles were computed for each 10-min file. Measured values of median noise in the selected frequency band were compared in the presence and absence of fin whale calls respectively. Two sub-samples were selected for this analysis: the first comprising all the 257 files data with the detection of fin whale calls (SB1, n = 257). The second comprised 257 randomly selected files, extracted from the recording pool, without validation of fin whale acoustic presence (SB2, n = 257). Since SB1 and SB2 follow a non-parametric distribution (Shapiro-Wilk test, *p-value* < 0.01), a two-tailed Mann-Whitney U test was performed using “IBM SPSS Statistics 20.0” [65]. This test allowed us to verify whether the median PSD values were significantly different when fin whale calls were detected (null hypothesis: SB1 and SB2 equal for *p-value* ≥ 0.01).

### Estimating the calls’ detection range

**Fig 3:**
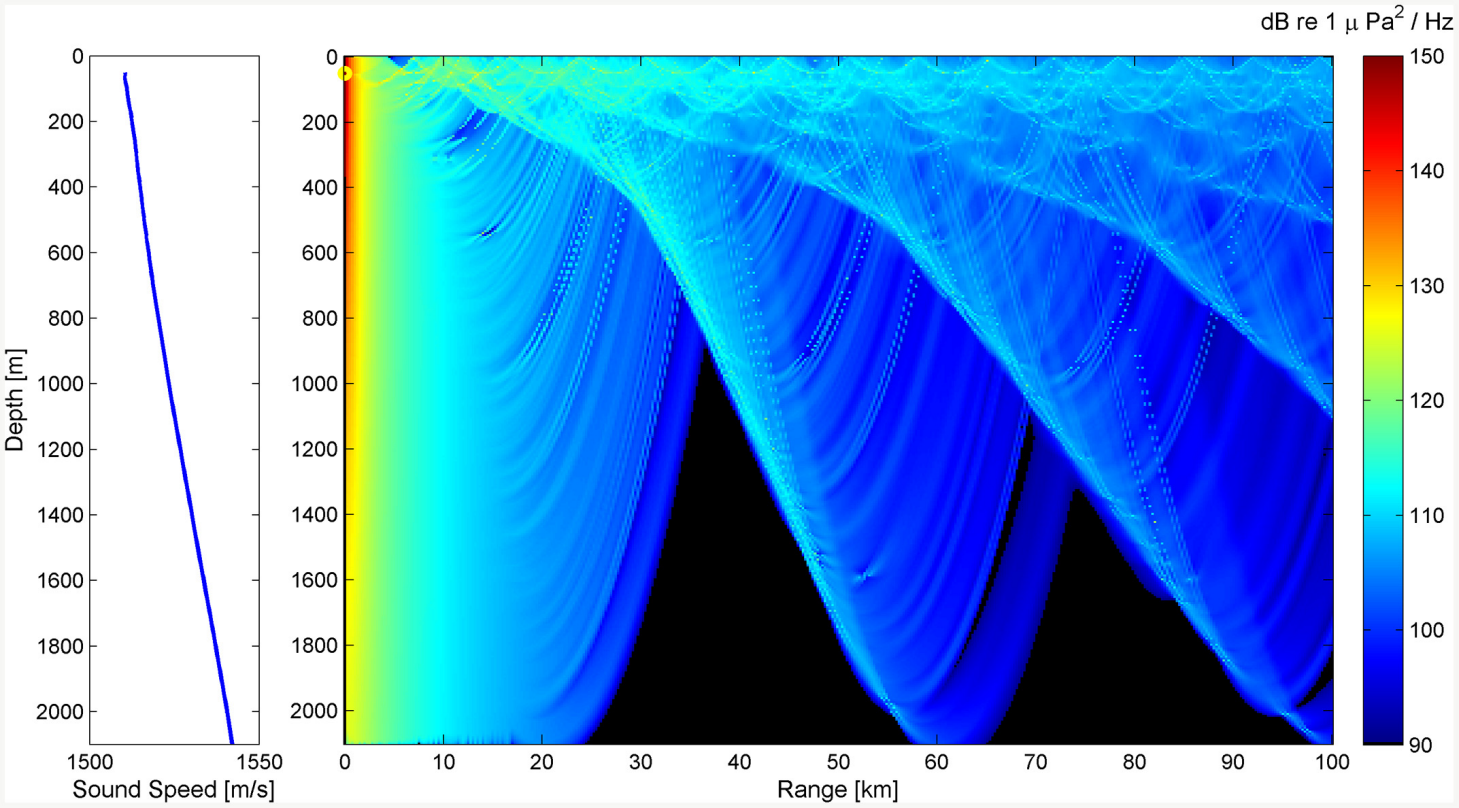
Transmission loss model. Results of BELLHOP incoherent transmission loss model of a 20 Hz signal emitted at a depth of 50 m (SL = 189 dB re 1 *μ*Pa at 1 m) [66]. The lower limit of the amplitude transmission is set to 90 dB (black area), which represents the baseline noise value at 20 Hz in the area.

In order to evaluate the typical range of detection of fin whale calls with the NEMO-SN1 low-frequency hydrophone, a simple sound propagation model was developed using the BELLHOP algorithm [66]. The model included attenuation due to geometric spreading and absorption processes related to the salts dissolved in the seawater. The Sound Velocity Profile (SVP) used in the model was obtained from CTD (Conductivity, Temperature and Depth) data acquired in previous campaigns in the Gulf of Catania using an MK-317 CTD from Idronaut [67]. The typical propagation range of fin whale calls (20 Hz) was calculated assuming that the sound source was located at a depth of 50 m, which is the typical depth at which fin whales produce calls [19, 44, 68, 69]. The most recent estimates of Source Levels (SL), obtained by deep-sea acoustic monitoring systems, indicate an average value of 189 ± 6 dB re 1*μ*Pa at 1 m [20, 21]. This value was selected as typical SL for both A and B call types. In Fig 3 the expected received amplitude along the water column is depicted as a function of distance from the source. Since the NEMO-SN1 observatory is located on the seafloor, at a depth of 2,100 m, the simulated sound amplitude reaches at a distance of about 25 km the value of 90 dB re 1 *μ*Pa, which is the estimated baseline noise at 20 Hz in the area.

### Ethics Statement

The NEMO-SN1 deep-sea cabled infrastructure was deployed off East-Sicily coasts with proper authorization issued from civil and military authorities at national, regional and local level: Marina Militare Italiana, AutoritÃ Portuale di Catania, Ministero dell’Ambiente and Direzione Marittima di Catania. The fin whale, *Balaenoptera physalus* (Mediterranean subpopulation), is included in the “IUCN Red List of Threatened Species” and listed as “vulnerable” [4]. Despite this, no approval was necessary to monitor the presence of fin whale in the area, since the detection technique applied (passive acoustic monitoring, using hydrophones deployed on the seabed) did not imply any interference or contact with the animals under study and no field studies were performed on the species.

## Results

### Fin whale acoustic presence

**Fig 4:**
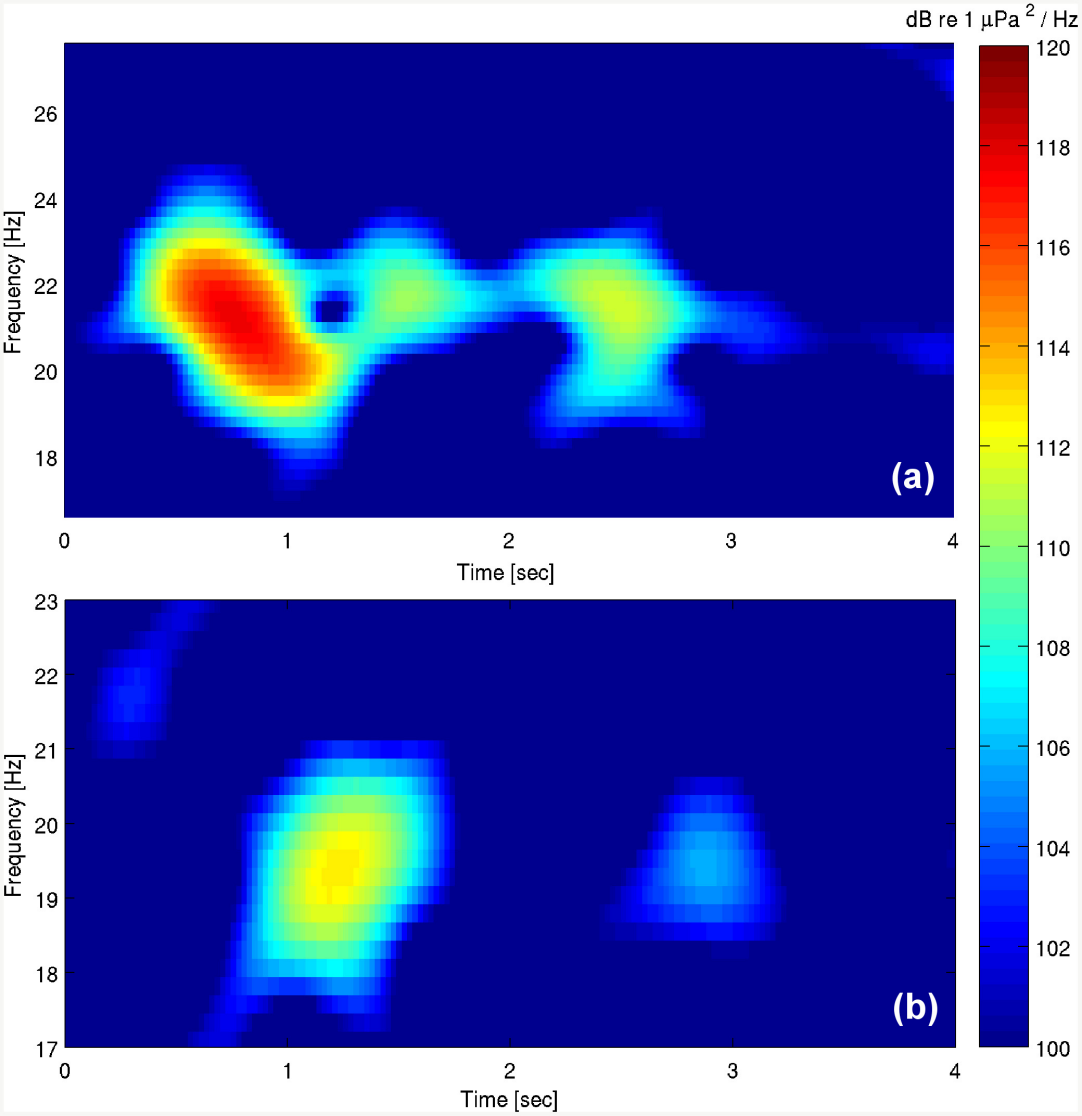
Observed fin whale call types. Spectrograms (8192 points FFT, 2048 points Hanning window, 97% overlap) of two typical calls of the Mediterranean fin whale. (a) type A or “classic pulse” and (b) type B or “back-beat” are here showed in detail.

**Fig 5:**
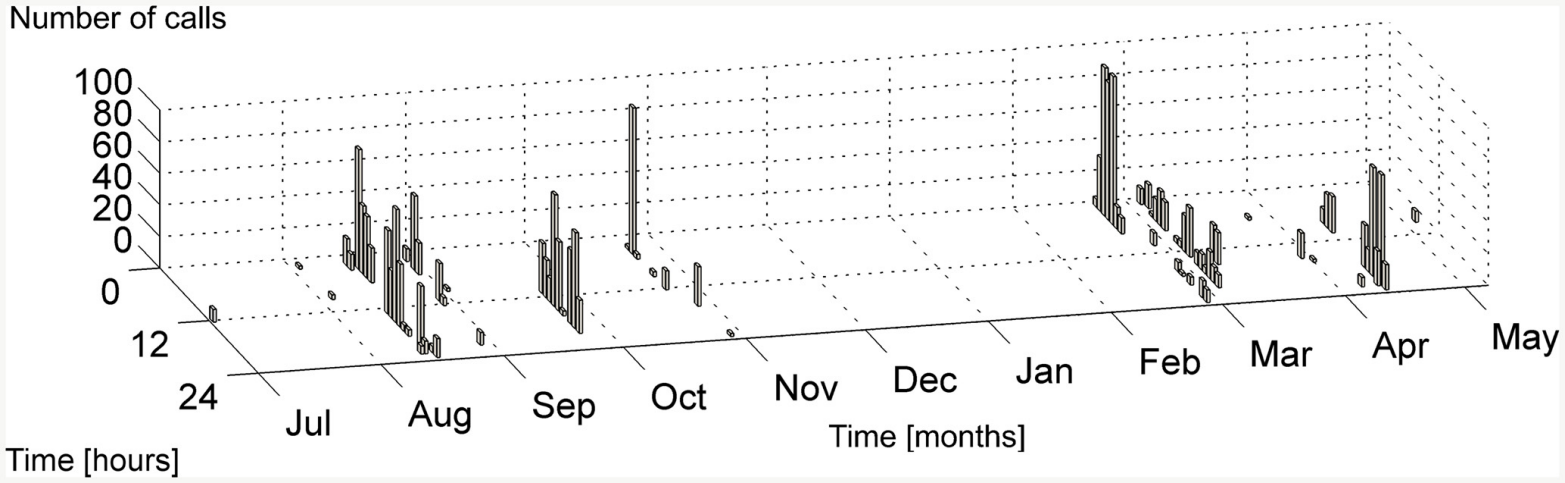
Time distribution of the detections. For each day of the recording months (x-axis), the number of calls is shown (z-axis) as a function of the hour in which the detection occurred (UTC time, 24-hour format) (y-axis).

**Fig 6:**
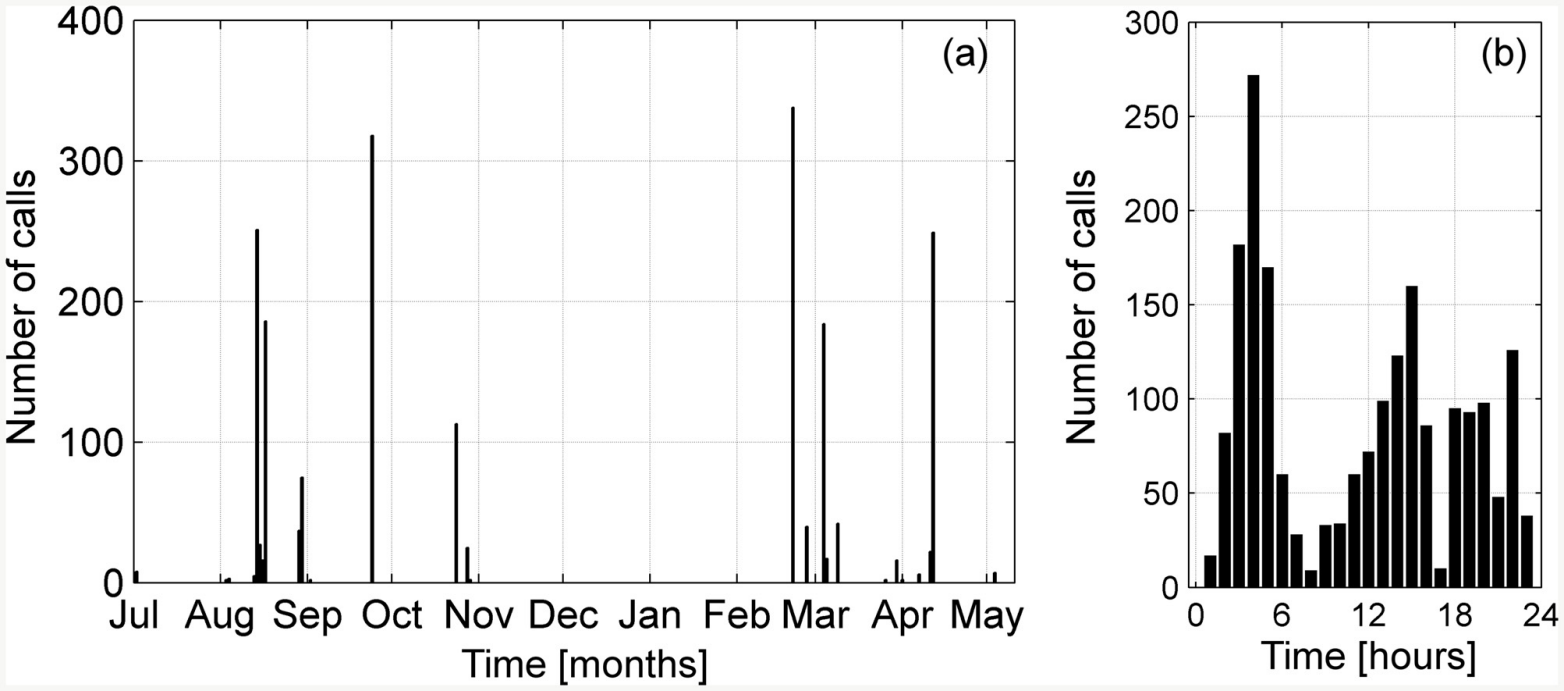
Daily and hourly distribution of the calls detected. (a) Total number of calls detected per day from 2^nd^ July, 2012 to 10^th^ May, 2013. (b) Total number of calls detected per hour in UTC time, 24-hours format.

**Fig 7:**
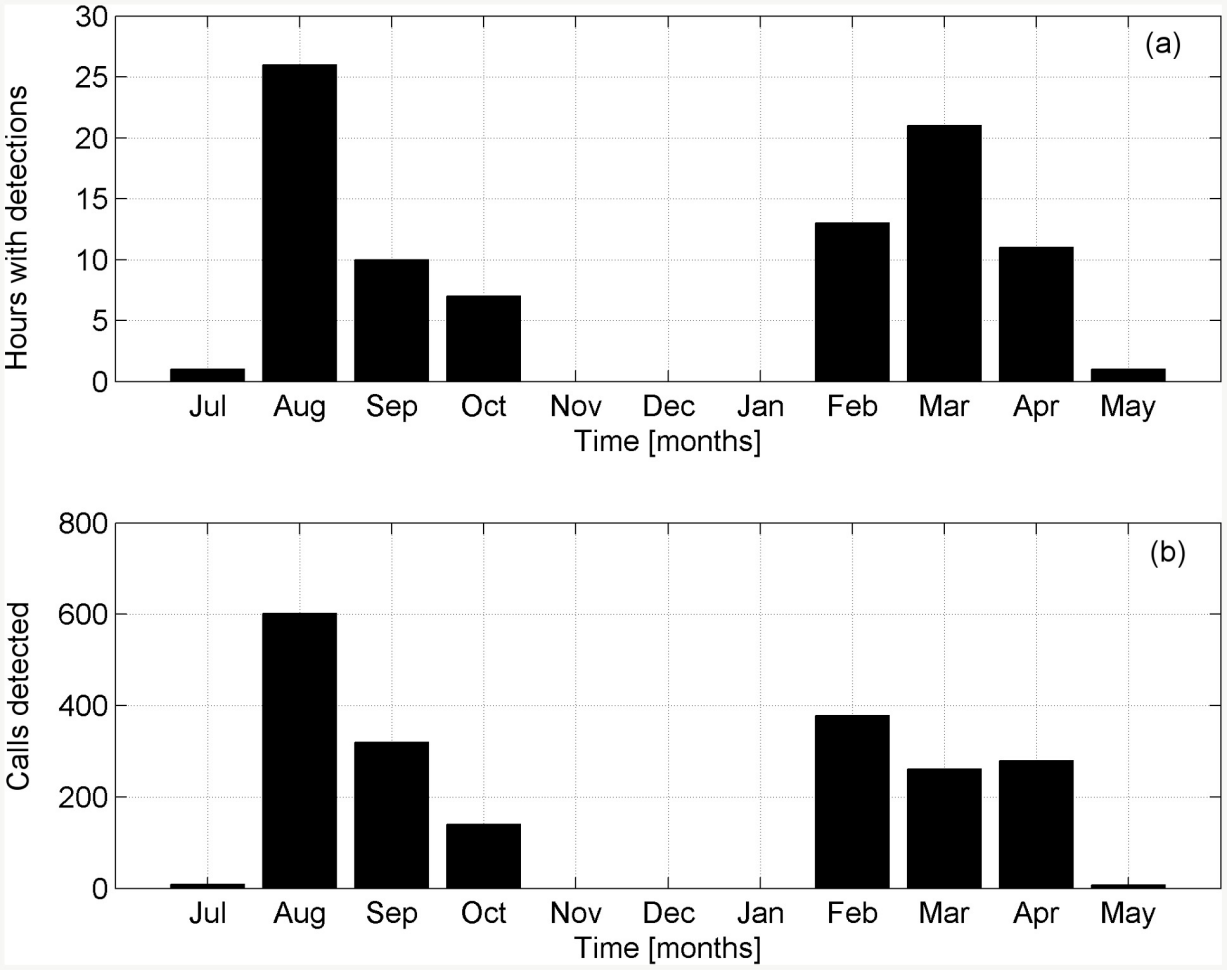
Fin whale detections per month. (a) Total number of hours with detections per recording month. (b) Total number of 20 Hz calls detected per recording month.

The analysis of about 7,200 recording hours (from 2012-07-02 to 2013-05-10) revealed the occurrence of fin whale calls in 90 detection hours (257 recordings of 10-min duration) (Table 1). Both types of the typical 20 Hz calls (A and B) (Fig 4a and 4b) associated with the Mediterranean subpopulation [2, 51] were noticed in 7 of the 10 months of continuous recording time. The time distribution of the calls as a function of the day of the month and of the hour of the day is illustrated in Fig 5 and in Table 1. Detections occurred in 27 different days, corresponding to about the 12% of the recording days, from July 2012 to October 2012 and from February 2013 to May 2013. No detections occurred between November 2012 and January 2013. The highest rates of calls per day were observed on the 2013-02-21 (338 calls, within 7 recording hours) and on the 2012-09-24 (440 calls, within 9 recording hours), as shown in Fig 6a and in Table 2. On the other hand, August 2012 was the month with the largest absolute number of detections (Fig 7). During this month, about 600 calls (Fig 7b) were detected in 26 recording hours (Fig 7a), spread over 9 recording days (Fig 6a, Table 2). In addition, August 2012 features the longest period of consecutive daily acoustic presence (5 consecutive days). This is shown in Fig 6a. Furthermore, the 24-hour distribution of all the calls detected is illustrated in Fig 6b. To evaluate the presence of diel patterns, the detection rates were grouped into the four diel periods (dawn, day, dusk and night). The distribution of the detections did not show any significant difference between the four periods (Kruskal-Wallis ANOVA, *p-value* > 0.05).

**Table 1:** Fin whale acoustic detections. The table reports per each recording month the number of analyzed days, days with detections and hours with detections.

Month	Analyzed days	Detection days	Detection hours
July, 2012	30	1	1
August, 2012	31	9	26
September, 2012	30	2	10
October, 2012	31	3	7
November, 2012	30	0	0
December, 2012	31	0	0
January, 2013	31	0	0
February, 2013	28	2	13
March, 2013	31	5	21
April, 2013	30	4	11
May, 2013	10	1	1
Total	313	27	90

**Table 2:** Distribution of fin whale acoustic detections within the recording dataset. Each row displays: the day with fin whale calls detected (Date); the number of hours with presence of fin whale calls per day (Hours); the number of 10-min long files with calls detected per day (Files); the total number of fin whale calls detected per day (Calls).

Date	Hours	Files	Calls
July 2^nd^, 2012	1	1	8
August 3^rd^, 2012	1	1	2
August 4^th^, 2012	1	1	3
August 13^th^, 2012	1	2	5
August 14^th^, 2012	8	25	251
August 15^th^, 2012	2	3	27
August 16^th^, 2012	2	2	16
August 17^th^, 2012	5	23	186
August 29^th^, 2012	3	5	37
August 30^th^, 2012	3	7	75
September 2^nd^, 2012	1	1	2
September 24^th^, 2012	9	34	318
October 24^th^, 2012	5	11	113
October 28^th^, 2012	1	4	25
October 29^th^, 2012	1	1	2
February 21^st^, 2012	7	27	338
February 26^th^, 2012	6	13	40
March 4^th^, 2013	15	40	184
March 5^th^, 2013	2	2	17
March 9^th^, 2013	2	7	42
March 26^th^, 2013	1	1	2
March 30^th^, 2013	1	4	16
April 1^st^, 2013	1	1	2
April 7^th^, 2013	1	2	6
April 11^th^, 2013	1	1	22
April 12^th^, 2013	8	29	249
May 4^th^, 2013	1	3	7

### Fin whale call detection and background noise

**Fig 8:**
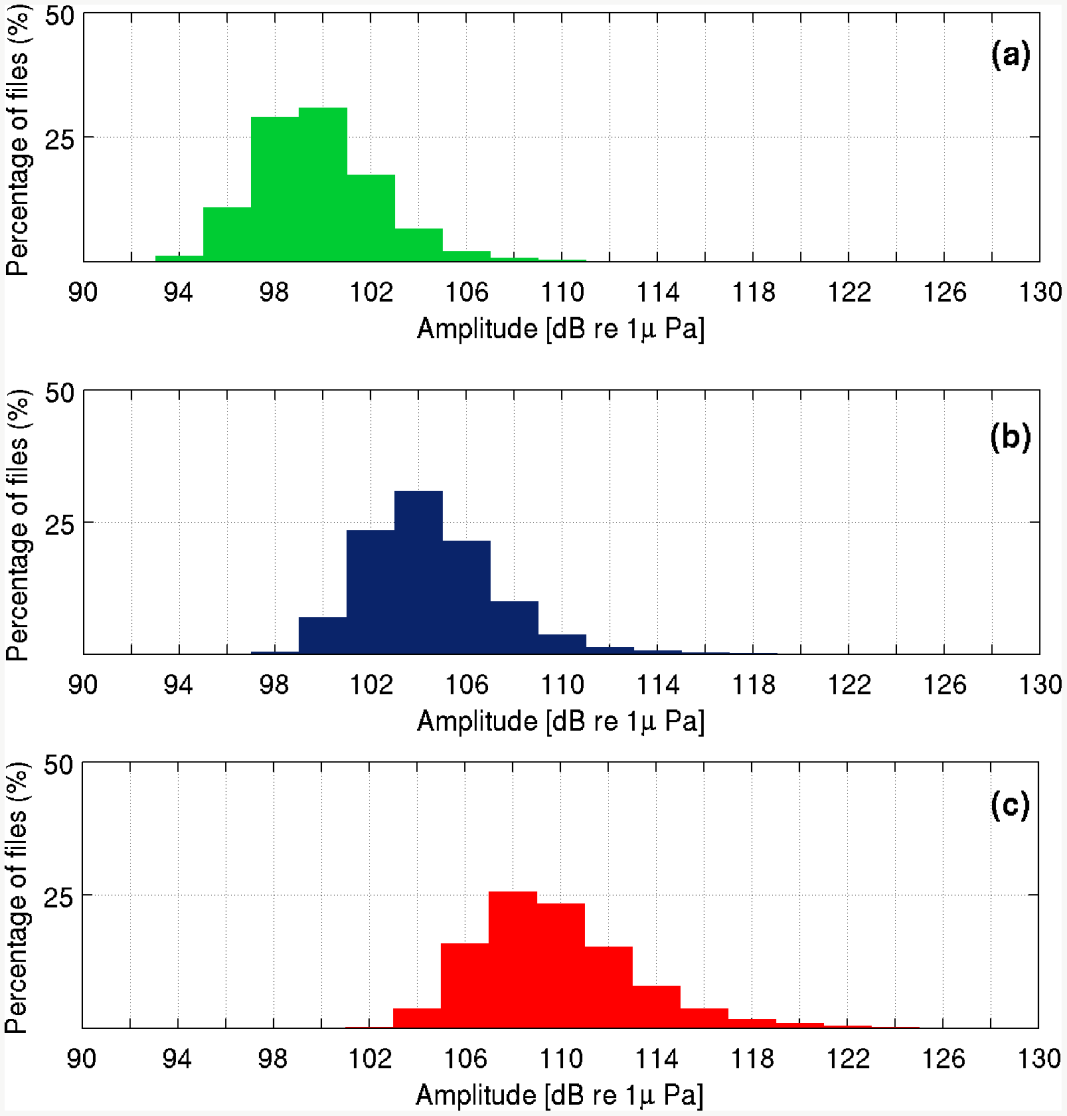
Background noise distribution. Distribution of the 5^th^ (a), the 50^th^ (median) (b), and the 98^th^ (c) percentiles of noise integrated within the 17.9–22.5 Hz frequency band, measured per each 10 minute file (2 dB resolution).

**Fig 9:**
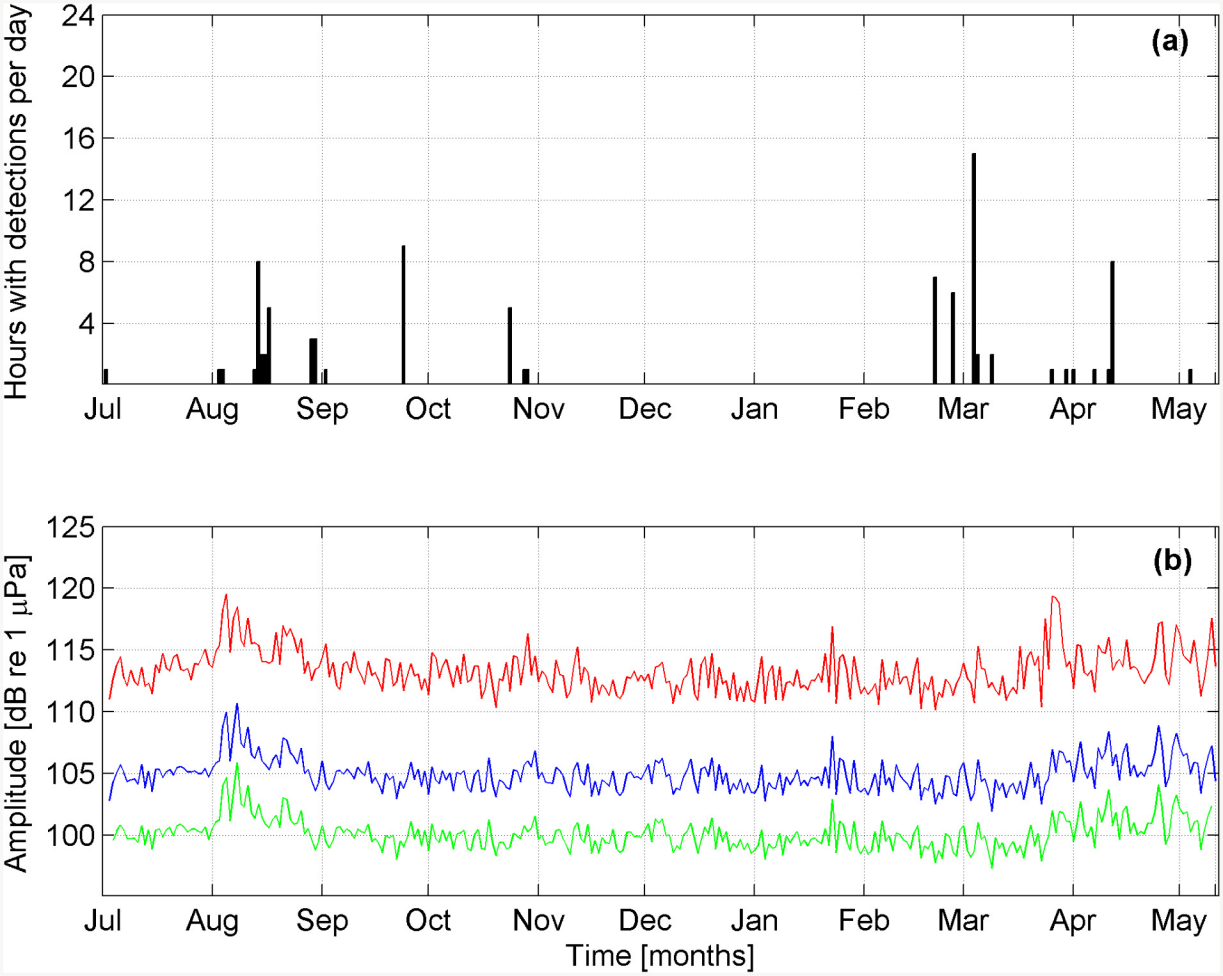
Fin whale daily acoustic presence and noise levels. (a) The total number of hours with detection of fin whale calls is shown per recording day, from 2^nd^ July, 2012 to 10^th^ May, 2013. (b) The 5^th^ (green line), the 50^th^ (blue line) and the 98^th^ (red line) percentiles of noise integrated in the 17.9–22.5 Hz frequency band measured per 10-min file and averaged per analyzed day.

The statistical analysis of background noise, performed on the whole data set, shows that the median value of noise (in the 17.9–22.5 Hz band) varies between 98 and 116 dB re 1*μ*Pa, per 10-min sample (Fig 8). As shown in Fig 9, the daily average of noise percentiles in the band considered varies slightly from day to day. Results of statistical analysis, performed on 10-min samples, indicate that the median of the noise level in samples where fin whale calls were detected was significantly lower than in the randomly chosen subset of 10-min samples used for the Mann–Whitney U-test (n = 508, z = −4.771, p < 0.001). By applying a transmission loss model to the samples, we estimated the typical detection range within a radius of about 20 km from the sensor (Fig 3), with a background noise level of 104 dB re 1 *μ*Pa. Such level corresponds to the typical value of median acoustic noise, occurring in the 30% of the recordings (Fig 8); the same model shows that when noise is 110 dB re 1 *μ*Pa, the detection range is limited to about 10 km (Fig 3). This value is exceeded in approximately 20% of the recordings (Fig 8).

## Discussion

The results, obtained analyzing about 10 months of continuously recorded data, provided for the first time valuable information on the presence of fin whales and their acoustic activity offshore Eastern Sicily. As proposed by several authors [12, 15, 51], the Ionian Sea could represent an important transit and aggregation area for at least a part of the Mediterranean population, which seasonally congregates in high productivity regions. In the past, the presence of the fin whales offshore Eastern Sicily was only monitored by means of sporadic visual observations [11, 14]. The lack of published records concerning the Ionian area, reported by Notarbartolo et al. [1], remains the main obstacle in assessing fin whale seasonal presence and distribution. Nevertheless, this work shows the effectiveness of continuous passive acoustic monitoring and it represents the first inter-seasonal investigation carried out in the area. Detected fin whale vocalization types were consistent with former observations conducted on the Mediterranean population [2, 51]. The annual pattern of these detections shows peaks in fin whale acoustic activity during spring and summer months, while no detections occurred between November 2012 and the end of February 2013. This paper also highlights the very high background noise level in the main frequency band of fin whale communication. The observations indicate that median noise levels around 20 Hz varied slightly around dramatically high values (98–116 dB re 1 *μ*Pa), throughout all the investigated months. This result, in agreement with previous studies, confirms that the Mediterranean acoustic habitat is characterized by high levels of anthropogenic noise, due to the high-density marine traffic, and that the values observed here are higher than in other ocean basins [17, 51, 52]. The typical detection range was estimated to be of about 20 km (with 104 dB re 1 *μ*Pa background noise) considering the position of hydrophone, placed just above the seafloor (about 2,100 m water depth). Furthermore, the transmission loss model showed that fin whale sounds may be received in the top 200 m of the water column from a distance greater than 100 km, at a Received Level (RL) higher than 100 dB re 1 *μ*Pa. In spite of this, the very top surface water layers are subject to daily and seasonal variations of sound transmission path, while the acoustic detection space of NEMO–SN1 is not affected by these variations, due to the high-depth position of the receiver and to the typical position of the source [19, 44, 68, 69]. The calls of the species were moreover more frequently detected in low background noise conditions. In this study we could not discern whether the observed variations in fin whale acoustic presence are due to variations of the available detection space, or if the animals were not emitting calls or avoiding the area in presence of high noise levels. Nevertheless, noise still affects the ability to detect them acoustically and to correctly estimate their presence. Despite this uncertainty, it has been shown that fin whale calls were irregularly detected offshore Eastern Sicily, in 7 out of about 10 months of continuous passive acoustic monitoring, with a 3-months gap from November to January. The observed detection trend fits well into the hypothesis that the species occurs seasonally offshore Eastern Sicily, from late winter to summer months, migrating towards other productive zones, such as the Lampedusa Island, from late autumn and for winter months. [11, 12, 14].

## Conclusions

A firm conclusion on the seasonal presence and movements of the fin whale in the Ionian Sea will be only possible monitoring the area for several years and including new receivers spread on a broader region. This also in the aim of obtaining new information such as the behavior and the abundance of the individuals occurring offshore Eastern Sicily [69–71]. Increasing the knowledge on the seasonal paths of the species in this area will allow to better understand the distribution of the Mediterranean fin whale population within the whole basin. Further studies should take into consideration the influence of environmental and anthropogenic factors on fin whale seasonal and inter-annual occurrence in the area. Among these factors, long and short term variations of background noise levels, but also variations in prey abundance and oceanographic parameters [52, 72, 73] could be relevant in developing conservation strategies. Nevertheless, the preliminary analysis of background noise levels presented in this article lays the foundation to study how background noise affects fin whale acoustic communication space [17] in the Ionian Sea.
